# Deficient DNA mismatch repair and persistence of SARS-CoV-2 RNA shedding: a case report of hereditary nonpolyposis colorectal cancer with COVID-19 infection

**DOI:** 10.1186/s12879-021-06500-1

**Published:** 2021-08-21

**Authors:** Farzana Haque, Patrick Lillie, Farhana Haque, Anthony Maraveyas

**Affiliations:** 1 Hull University Teaching Hospital NHS Trust, Castle Road, Cottingham, Hull, HU16 5JQ UK; 2 Hull York Medical School, University of Hull, Allam Medical Building, Hull, HU6 7RX UK; 3 Lincoln Medical School, University of Lincoln, Lincoln, LN6 7DL UK

**Keywords:** Case report, Mismatch repair, SARS-CoV-2, Hereditary nonpolyposis colorectal cancer

## Abstract

**Background:**

Several independent risk factors have been reported to influence viral shedding following COVID-19 infection, but the influence of host-related molecular factors has not yet been described. We report a case of a cancer patient with Lynch syndrome (hereditary nonpolyposis colorectal cancer, HNPCC) who manifested SARS-CoV-2 PCR (polymerase chain reaction) positivity for at least 54 days after contracting mild COVID-19 illness. We propose that deficient mismatch repair (MMR) may play a role in the prolonged SARS-CoV-2 RNA shedding.

**Case presentation:**

A patient with Lynch syndrome was under surveillance for metastatic adenocarcinoma after completing palliative chemotherapy in October 2019. Between the period of April 2020 to June 2020, he was admitted multiple times to address several clinical needs mainly related to his underlying malignancy. These included progressive disease observed in the aortocaval lymph nodes leading to recurrent episodes of upper gastrointestinal bleeding, dehydration resulting in acute kidney injury and a short-lived episode of pyrexia. A SARS-CoV-2 PCR of the nasopharyngeal swab (NPS) was positive at his initial admission with mild COVID-19 symptoms. He remained positive on subsequent admissions when tested routinely for SARS-CoV-2 without demonstrating any apparent clinical features of COVID-19 infection.

The MMR pathway, a component of DNA damage response (DDR), is impaired in Lynch syndrome due to an inherited genetic mutation. This pathway is also required for viral clearance from the host cells following certain RNA viral infections like influenza virus and other coronaviridae. Here we provide a current understanding of the importance of DDR deficiencies in the clearance of RNA virus and suggest how this may play a similar role in the clearance of COVID-19, as evident in our case that demonstrated persistent positivity.

**Conclusion:**

The importance of understanding the scientific basis of extended viral shedding during the COVID-19 pandemic is now centre-stage in the establishment of robust track and trace services to allow the recovery and function of societies and economies. This patient with Lynch syndrome recovered from infection but had prolonged viral positivity, which might merit further investigation to better understand the effect of this condition on infection duration and outcome.

## Background

Preliminary reports show that the median duration of shedding for severe acute respiratory syndrome coronavirus 2 (SARS-CoV-2) is 20 days (range 8–37 days) [1]. Although, it can last longer for the hospitalized patients with severe COVID-19 infection [2–4]. Several independent risk factors have been identified to be responsible for the prolonged SARS-CoV-2 ribonucleic acid (RNA) viral shedding [5–7], which can be up to 34 days (IQR 24–40 days). These include obesity, age ≥ 65 years, male sex and invasive mechanical ventilation. It is unknown whether viral or host-related molecular mechanisms are involved in the efficiency of SARS-CoV-2 RNA clearance by the host cells. However, literature exists on the contribution of the DNA MMR (mismatch Repair) pathway in the antiviral response to other RNA viruses, e.g., influenza, coronavirus [8]. Herein, we report a case of hereditary nonpolyposis colorectal cancer (HNPCC) which, after contracting mild COVID-19 infection, manifested prolonged polymerase chain reaction (PCR) test positivity for SARS-CoV-2. We propose that deficient-MMR (dMMR) may have a role in the persistent shedding of SARS-CoV-2 RNA.

## Case presentation

The case is of a 69-year-old man with HNPCC (Lynch Syndrome, mutation in exon 15 of hMSH2), with a history of pancolectomy and Ileo-rectal anastomosis in 2004, completion proctectomy and ileostomy for pT1 adenocarcinoma in the rectal remnant in 2012 and nephroureterectomy for pT1 grade 3 transitional cell carcinoma of the left ureter in 2014. In 2016, he received radical external beam radiotherapy for T3aN0M0 prostate adenocarcinoma.

On January 17th, 2018, he underwent a Whipple’s procedure and a small bowel resection for synchronous pT3aN1(1/21) M0 adenocarcinoma of the ampulla and pT3 adenocarcinoma of the duodenum. He was left with a high output stoma. His body mass index (BMI) was 25, he was on anticoagulants for recurrent venous thromboembolism (VTE) and had no other comorbidities.

Surveillance imaging in June 2019 demonstrated metastases to the aortocaval lymph nodes. He received FOLFOX chemotherapy until October 2019 and subsequently remained stable.

On April 4th, 2020, he presented with recurrent bleeding episodes per ileostomy and a 2-day history of fever, lethargy and dry cough. His highest recorded temperature was 37.5 °C, he was not breathless and his oxygen saturation was 99% on air. His CURB-65 score was 3 [Urea 8.8 mmol/l, diastolic blood pressure (BP) 60 mm (Hg) and age > 65], and he was anaemic (haemoglobin 80 g/l), leukopenic (WBC 3.3 × 10^9^/L) and lymphopenic (0.75 × 10^9^/L). His coagulation screen was normal and his blood type was A-positive. He tested positive (cycle threshold [CT] 16.25) for SARS-CoV-2 PCR nasopharyngeal swab (NPS). Computed tomography of the chest/abdomen/pelvis showed peripheral patchy ground-glass appearance, primarily in the right lower lobe of the lung. There was evidence of disease progression with enlarged aortocaval nodes that were now invading the adjacent Roux limb. He received red blood cell transfusions, intravenous fluids and antibiotics and his anticoagulation was withheld. He continued to manifest melena with tachycardia (100/min) and hypotension (BP 96/56 mmHg), and therefore received a further transfusion, tranexamic acid for two days and palliative haemostatic radiotherapy (20 Gy in 5 fractions) to the aortocaval mass. A SARS-CoV-2 PCR NPS on day 24 was positive (CT 34.6), and once his symptoms improved he was discharged.

He was readmitted 3 weeks later with dehydration, reduced stoma output and acute kidney injury. There were no symptoms of COVID-19; however, his NPS was still positive (CT 33.4) for SARS-CoV-2 (Day 54). He improved with supportive treatment and was discharged after five days.

In June 2020, he was seen for short-lived pyrexia. The chest X-ray was normal and no apparent infective cause was found. SARS-CoV-2 PCR (day 63) from NPS was negative at this time. His COVID-19 total antibody level was consistent with the exposure to SARS-CoV-2.

## Discussion and conclusion

The median duration of viral shedding is 2–3 weeks in mild COVID-19 disease [1–4]. In this case, although he had recovered from mild COVID symptoms, it persisted for at least 54 days. Besides being a male and older than 65, he had no risk factors for extended SARS-CoV-2 RNA shedding.

Several genetic factors have been identified which could be used in the clinical stratification and management of patients infected with the SARS-CoV-2 combined with other cellular and clinical parameters. These genetic factors include ABO gene, variants of SLC6A20, ERMP1, FCER1G and CA11, low expression of *IFNAR2*, and high expression of *TYK2* HLA DQA1_509. [9–13]. These genetic associations signify an increased risk or correlation with severity of infection; however, any association with viral clearance has not yet been reported.

HNPCC is an autosomal dominant genetic condition of inherited mutations that causes impaired/deficient DNA mismatch repair (dMMR). Mutation in the MMR gene can also be found in uterine endometrial carcinoma, colon adenocarcinoma, stomach adenocarcinoma, rectal adenocarcinoma, adrenocortical carcinoma, uterine carcinosarcoma, cervical cancer, Wilms tumour, mesothelioma, and oesophageal carcinoma [14, 15].

**Fig. 1:**
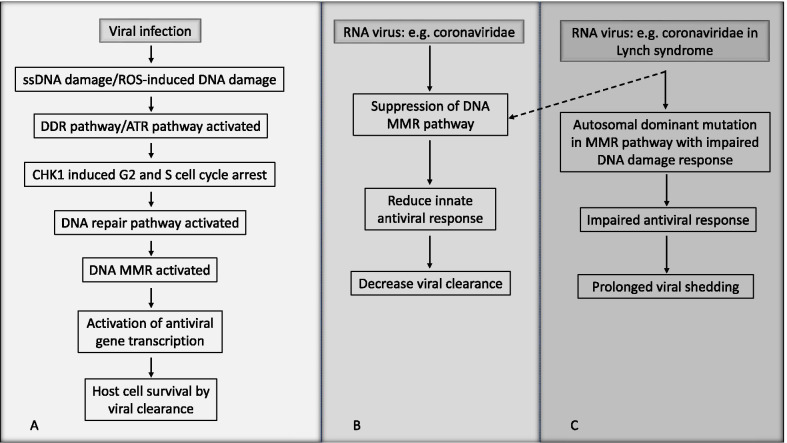
Cellular viral clearance response after viral infection. Schematic representation of cellular viral clearance response after viral infection (**A**), in case of coronaviridae (**B**) and this patient with Lynch syndrome (**C**). ss (single strand), ROS (reactive oxygen species), DDR (DNA damage repair), ATR (ATM and Rad3 related), CHK (Checkpoint), MMR (mismatch repair)

The DNA MMR pathway is a component of DNA damage response (DDR). It is required for innate cellular antiviral response and control of cellular fate following Influenza A Viral infection [8] (Fig. 1). This pathway prevents the accumulation of oxidative DNA lesions in the antiviral gene foci. It contributes to non-lytic viral clearance and cell survival in club cells, a subset of epithelial cells in the lungs [8]. A higher level of DNA MMR activity allows repair of virus-induced damage and facilitates the transcriptional expression of these antiviral genes [8].

The RNA viruses can induce significant DNA damage/genetic instability in the host cells during their life cycle; for example, Human immunodeficiency virus 1, Human T-cell lymphotropic virus, Hepatitis C virus (HCV), Infectious bronchitis virus, Influenza A virus (IVA), Chikungunya virus, Sindbis virus, La Crosse virus, Rift valley fever virus and Avian Reovirus [16]. They manipulate components of the DDR pathway, which allows their pathogenesis and propagation [16]. Some RNA viruses (e.g., IAV, HCV and alphaviruses) activate DNA damage response and cause downregulation of the DNA MMR pathway [8, 17]. Chambers et al. found that while the IAV generally leads to reduction of DNA MMR in the infected host cells, club cells have the unique ability to maintain normal levels of DNA MMR and thereby survive the infection. Suppression of the DNA MMR pathway in the club cells with recombinant IVA strain prevents club cell survival and causes an increase in the severity of viral disease in vivo [8]. This study points to the role of the MMR pathway in the promotion of genes responsible for viral clearance in the infected cells (Fig. 1). Therefore, a connection may exist between dMMR and persistent positivity for viral shedding following SARS-CoV-2 infection, where the host repair mechanism is affected following oxidative damage and impaired MMR, resulting in reduced viral clearance.

The long or short-term clinical significance of our observation is noteworthy. Although this patient manifested only mild COVID-19 disease and mounted an antibody response within an expected time course, he had shed the virus for 54 days. We have not found any ongoing post-COVID morbidity separate from the symptoms of his underlying malignancy and treatment.

The limitation in our case was the availability of NPS PCR as the sole diagnostic test to assess prolonged shedding. Testing the level of total antibody became available subsequently, which was checked to confirm the exposure.

This patient with Lynch syndrome recovered from infection but had prolonged viral positivity, which may merit further investigation to better understand the effect of this comorbidity on infection duration and outcome.

## SARS-CoV-2 PCR assay

Roche Cepheid assay was used for nasopharyngeal swabs tested for SARS-COV-2.

## Data Availability

Fully anonymised data are freely available on the case report and will be provided upon request.

## Abbreviations

ATR: ATM and Rad3 related
BP: Blood pressure
CHK: Checkpoint
COVID-19: Coronavirus disease 2019
CURB-65: Confusion, blood urea nitrogen, respiratory rate, blood pressure, age 65 or older
DNA: Deoxyribonucleic acid
dMMR: Deficient mismatch repair
DDR: DNA damage pathway
FOLFOX: Folinic acid, fluorouracil, oxaliplatin
Gy: Standard dose gray
Hb: Haemoglobin
HCV: Hepatitis C virus
HNPCC: Hereditary nonpolyposis colorectal cancer
IAV: Influenza A virus
IQR: Inter quartile range
M: Metastases
MMR: Mismatch repair
N: Lymph node
NPS: Nasopharyngeal swab
PCR: Polymerase chain reaction
ROS: Reactive oxygen species
RNA: Ribonucleic acid
SARS-CoV-2: Severe acute respiratory syndrome coronavirus 2
Ss: Single strand
T: Tumour
WBC: White blood cell
